# On the Surgical Treatment of Renal and Ureteral Calculi with Report of 82 Cases

**Published:** 1913-03

**Authors:** 


On the Surgical Treatment of Renal and Ureteral Calculi with Report of 82 Cases.—Dr. W. P. Kouzuetzky, St. Petersburg (*Zent. fur Chir.*, November 23, 1912). The author describes a conservative method of removing stones, namely, by entering through the posterior surface of the pelvis, leaving the upper pole and anterior surface of the kidney covered with the pernephine fat. He advises separate incisions for nephrotomy should this be required, because of the size and positions of the stones. He mentions the dangers from haemorrhage, but fails to note the wire method of nephrotomy by which this danger is so largely diminished.

